# Cardiovascular Disease Recognition Based on Heartbeat Segmentation and Selection Process

**DOI:** 10.3390/ijerph182010952

**Published:** 2021-10-18

**Authors:** Mehrez Boulares, Reem Alotaibi, Amal AlMansour, Ahmed Barnawi

**Affiliations:** 1 Information System Department, Computing College, King Abdulaziz University, Jeddah, Makkah 21589, Saudi Arabia; ralotibi@kau.edu.sa (R.A.); aalmansour@kau.edu.sa (A.A.); ambarnawi@kau.edu.sa (A.B.); 2 Research Laboratory of Technologies of Information and Communication and Electrical Engineering (LaTICE), Higher National School of Engineers of Tunis (ENSIT), University of Tunis, Tunis 1008, Tunisia

**Keywords:** CVD, heart sounds, PCG, denoising, segmentation, deep learning, convolutional neural network

## Abstract

Assessment of heart sounds which are generated by the beating heart and the resultant blood flow through it provides a valuable tool for cardiovascular disease (CVD) diagnostics. The cardiac auscultation using the classical stethoscope phonological cardiogram is known as the most famous exam method to detect heart anomalies. This exam requires a qualified cardiologist, who relies on the cardiac cycle vibration sound (heart muscle contractions and valves closure) to detect abnormalities in the heart during the pumping action. Phonocardiogram (PCG) signal represents the recording of sounds and murmurs resulting from the heart auscultation, typically with a stethoscope, as a part of medical diagnosis. For the sake of helping physicians in a clinical environment, a range of artificial intelligence methods was proposed to automatically analyze PCG signal to help in the preliminary diagnosis of different heart diseases. The aim of this research paper is providing an accurate CVD recognition model based on unsupervised and supervised machine learning methods relayed on convolutional neural network (CNN). The proposed approach is evaluated on heart sound signals from the well-known, publicly available PASCAL and PhysioNet datasets. Experimental results show that the heart cycle segmentation and segment selection processes have a direct impact on the validation accuracy, sensitivity (TPR), precision (PPV), and specificity (TNR). Based on PASCAL dataset, we obtained encouraging classification results with overall accuracy 0.87, overall precision 0.81, and overall sensitivity 0.83. Concerning Micro classification results, we obtained Micro accuracy 0.91, Micro sensitivity 0.83, Micro precision 0.84, and Micro specificity 0.92. Using PhysioNet dataset, we achieved very good results: 0.97 accuracy, 0.946 sensitivity, 0.944 precision, and 0.946 specificity.

## 1. Introduction

Sudden heart failure caused by cardiovascular diseases (CVDs) is one of the top causes of death globally. It causes about 17.3 million deaths per year, an amount that is estimated to rise to more than 23.6 million by 2030 according to the latest WHO report [1]. Moreover, it causes 45% of deaths in Europe [2], 34.3% in America [3], and more than 75% in developing countries [4]. In other words, due to unhealthy lifestyle, unavailability, financial or even carelessness constraints, persons neglect regular heart screening, which can favor the CVDs. Cardiovascular problems are considered as a potential medical emergency and must be detected without delay [5]. Earlier diagnosis of CVDs helps patients to decrease considerably the heart failure condition [6].

CVD diagnosis can be done by using the widely known auscultation methods based on stethoscope, phonocardiogram, or echocardiogram. A cardiologist expert could use phonocardiogram (or PCG) to visualize the recorded heart sound during a cardiac cycle based on a phonocardiograph device [7,8]. Also, they can use an echocardiogram (average cost of 1500 as per current cost [9]) to visualize the heart beating and blood pumping. Using a stethoscope, the cardiologist listens to the patient heart sound and tries to find out clues of unusual heart sound (murmurs), which is symptomatic of cardiac abnormalities. The recorded heartbeat sounds different between a normal heart sound and an abnormal heart sound as their PCG signal differs significantly from each other with respect to time, amplitude, intensity, homogeneity, spectral content, etc. [10].

Roughly, all of these heart screening procedures are expensive and require a lot of experience. As stated previously, auscultation requires an experimented cardiologist to obtain an accurate diagnosis [3]. According to some research, medical students and primary care physicians can reach only 20 to 40% accuracy in the heart screening process [11,12,13], and roughly 80% can be achieved when conducted by expert cardiologists [11,13]. In other words, there is a lack of a reliable solution for earlier diagnosis of CVDs.

Developing an accurate, accessible, and easy-to-use solution enables the democratization of the early heart screening, which can significantly help patients to stabilize or even to heal cardiovascular disease. Therefore, the PCG heart screening is considered a high-potential research topic that will expand and develop in the near future [11,13]. Many of the existing research work generally focuses on automatic cardiac auscultation based on classical machine learning methods [14,15] and deep learning models [16,17].

Relying on these ascertainments, this research aims at proposing a reliable CVD screening based on PCG signal classification. Particularly, an automatic method for PCG heart sounds analysis and classification, which is useful to detect heart pathology in clinical applications. The main contribution of our work concerns the proposition of a new and powerful preprocessing approach based on: infinite impulse response (IIR) filter for automatic noise deletion, an automatic powerful heart cycle segmentation (HCS) method based on envelop detection using Daubechie’s wavelet decomposition, a new HCS segment selection approach based on PCG feature clustering relaying on Gaussian mixture model (GMM). This new preprocessing approach is experimented on both Pascal and PhysioNet datasets with an extensive experimental study based on 17 convolution neural network (CNN) pretrained and fine-tuned models for the automatic PCG disease classification.

This paper is laid out as follows: Section 2 presents related work of existing methods, then Section 3 introduces the proposed model. The experiment setting and implementation are described in Section 4. Section 5 discusses the experimental results. Section 6 concludes the paper and indicates future and related research directions.

### Contributions

This research focuses on the e-health field and aims in providing a PCG classification approach that may help to detect earlier heart abnormalities. Our aim is to design and optimize an accurate algorithm to recognize the signatures of normal, murmur, and extrasystole heart rhythms using available experimental dataset. In this contribution, we focus on supervised machine learning techniques with the aim of extracting the signatures that identify normal, murmur, and extrasystole PCG signal. Our main contribution concerns the proposition of a new and powerful preprocessing approach that involves: IIR filter for automatic noise deletion; an automatic powerful Heart Cycle Segmentation (HCS) method based on envelop detection using Daubechies wavelet decomposition; a new HCS segment selection approach based on PCG feature clustering relaying on Gaussian mixture model (GMM), and an extensive experimental study based on 17 CNN pretrained and fine-tuned models for the automatic PCG disease classification.

## 2. Related Work

A substantial amount of research studies was presented towards the identification and classification of PCG signal, i.e., a digital heart sound signals recorded through an electronic stethoscope. Processing and analyzing PCG signal is based on solving three main challenges towards fully automatic heart sound identification and classification.

The first is preprocessing and PCG signal denoising to detect the noncardiac sounds. In this step, the additional noise is removed or reduced, and heart sounds are enhanced. This is usually achieved by removing some undesired frequencies or frequency bands in the signal, a process known as filtering.

The second challenge is heart sound segmentation, which is used to localize the main heart sound components. In this step, the heart sound signal is split into the following heart cycles: first heart sound (S1), the systolic period (siSys), second heart sound (S2), and the diastolic period (siDias). In the literature, there are several possible approaches to segment PCG signal. One of the used approaches is to identify the time instant and duration of the S1 and S2 heart sounds, by using some sort of a peak-picking algorithm. Advanced approaches apply temporal statistical models to search for the most likely hidden state sequence according to a set of observations.

The third challenge is feature extractions and classification of PCG signal into normal and abnormal heart sounds classes. In this step, researchers usually apply standard procedure that consists of the following steps: (1) extracting the features from the PCG signal, (2) feeding the selected classifier with the extracted features, and (3) finally, the classifier algorithm infers the presence or not of abnormal heart sounds.

Different survey papers discussed the PCG signal analysis challenges. A survey done by Meziani et al. discussed the analysis of different PCGs signals using wavelet transform-based methods (WT) only [18]. Another review was done by Chakrabarti et al. where the authors compared different methodologies used in the PCG signal analysis. Based on their comparative study, the authors suggested that empirical mode decomposition (EMD) is better suited for noisy PCG signals. In addition, they suggested the use of hybrid machine learning classifiers to improve the classification results [19].

Nabih et al. [20] reviewed research papers between 2004–2016 that cover intelligent computer-aided diagnosis (CAD) systems based on PCG signal analysis. They concluded that large databases are needed for use with different machine learning classifiers to improve the heart sounds classification accuracy. Also, they suggested to look on deep for more effective methods to reduce the heart sound signals noises.

### 2.1. PCG Signal Preprocessing, Denoising, and Enhancing

In the process of collecting the heart sounds recording, it is often disturbed by external and internal noisy sources such as chest movements, respiration sounds, muscle contraction, external noise from the surrounded environment, etc. All these noises may change the characteristics of recorded PCG signal and can make the analysis more difficult. Therefore, it is important to use appropriate denoising algorithm on PCG signal before any further analysis. PCG signal denoising is generally achieved through the utilization of suitable filter, most commonly infinite impulse response (IIR) or finite impulse response (FIR), to separate the PCG signal from the attached noises as a simple denoising method [21].

Kwak and Kwon [5] applied the Wiener filter to reduce the background noise, while Dewangan [22] developed an adaptive filter that can remove the noise from the signal using least mean square (LMS) algorithm. In [23], PCG signals were denoised using the maximally flat magnitude (Butterworth) filter. The authors in [24,25,26,27] applied wavelet transformation (WT), a well-known denoising technique to identify true PCG signal components. Another PCG signal denoising method can be achieved via EMD, where complicated data are decomposed into a finite-small number of components [28]. A combined multilevel singular value decomposition (SVD) and compressed sensing method is also proposed by [29] for PCG signal noise removal. Moreover, in the [30], PCG signal denoising technique was proposed based non-negative matrix factorization (NMF) and adaptive contour representation computation (ACRC).

### 2.2. PCG Signal Segmentation

Heart sounds segmentation is a fundamental step in PCG signal analysis. In this step, the locations of S1 (beginning of the systole) and S2 (end of the systole) heart sounds in a PCG signal are identified. Heart sounds are created by blood flow and vibrations of tissues during the cardiac cycle, and transient heart sounds can be classified into four heart sounds (S1, S2, S3, and S4). In general, only the first S1 and the second S2 heart sounds can be called as the main primary heart sounds, and the cardiac cycle can then be estimated according to the locations of S1 and S2. Certain variations over S1 and S2 properties such as their duration or intensities can be considered as the primal signs of cardiac anomalies.

For PCG signal segmentation, there exists various prior research works that proposed different techniques: firstly, the envelope-based method, which is one of the popular approaches in PCG segmentation. Choi and Jiang made a comparative study about the most used envelope-based methods: Shannon energy, Hilbert transform, and the casdiac Sound characteristic waveform (CSCW) [31]. Shannon energy and entropy envelope was used by [25,26,32,33,34,35,36]. Other techniques use envelope extraction based on WT to gain the frequency characteristics of of S1 and S2 sound components [15]. Various research studies used different envelope extraction methods for segmentation including Hilbert phase envelope [33], ensemble empirical mode decomposition (EEMD) [37], Hilbert transform [38,39,40], and autocorrelation [41,42].

Recently, methods such as a hidden Markov model (HMM) and a hidden semi-Markov model (HSMM) were used [43,44] for PCG segmentation. Gamero and Watrous [44] suggested the use of HMM to identify the S1 and S2 sounds. They used a topology combining two separate HMMs to model the Mel-Frequency Cepstral Coefficients (MFCC) of both systolic and diastolic intervals, respectively. The method achieved a sensitivity of 95% and positive predictivity of 97%. Schmidt et al. [43] proposed a method that extracts a range of features that are then used to train a duration-dependent HSMM to segment the PCG heart signals. Moreover, Logistic Regression-HSMM-based algorithm [45] is considered one of the most advanced method that achieved reasonable results in heart sound segmentation. Springer et al. [45] used the HSMM with the modified Viterbi algorithm to identify the start and end state of the PCG heart sound signal. The proposed method achieved an average F1 score of 95.63% on the testing dataset.

### 2.3. PCG Signal Feature Extraction and Classification

Feature extraction is a key step in PCG signal analysis as extracting the correct features is the basis for a successful heart sounds classification. Most of the extracted features for PCG heart signal are computed mainly using time, frequency, and statistical measures. A list of the most used features are as follows: heart rate, duration of S1, S2, Systole or Diastole, total power of the PCG signal, zero crossing-rate, MFCC, WT, Linear Predictive Coding (LPC) coefficients, and Shannon entropy. After extracting PCG signal features, the next step is to select suitable classifier to perform the classification process. Various machine learning algorithms were proposed by researchers to complete the PCG heart signal classification, such as artificial neural network (ANN), support vector machine (SVM), K-nearest neighbors (KNN), and other blended classification methods.

ANN is one of the most widely used machine learning-based approaches for classification. However, there is relatively little work done on deploying this method in heart signals identification. Eslamizadeh and Barati [46] used the ANN for heart disease classification. Continuous wavelet transform (CWT) with Morlet wavelet function were used to extract primary heart sounds S1 and S2 from the PCG signal. Features such as maximum amplitude were first normalized and then used by the ANN classifier to detect the murmur of heart sound signals.

Another successful machine learning algorithm that used mostly for heart sounds classification is SVM. Zheng et al. [15] used SVM to identify automatically the coronary heart diseases. Wavelet decomposition methods were utilized firstly on the PCG signal, and then the total energy and the sample entropy of each sublevel are used as input features for the SVM classifier. A classification accuracy of 97.17%, with a specificity of 98.55% and a sensitivity of 93.48%, were reported using the proposed method.

Research done by Kang et al. [47] also used SVM and ANN classifiers to detect Still’s murmur in children. They used the following features for classification: time domain features, including the average Shannon energy and envelope detection in addition to the frequency domain features, specifically the spectral width and peak frequency of the main heart sounds S1 and S2. They achieved to 84–93% sensitivity and 91–99% specificity using the proposed classification method. On other hand, Deng and Han [48] reached to accuracy equal to 91% using SVM classifier and autocorrelation features such as the sub-band autocorrelation function. Discrete wavlet transform (DWT) was used to identify the sub-band envelopes derived from the sub-band coefficients of PCG signal which then was used to extract autocorrelation features. Later, these features were fused using diffusion maps to get unified features and fed to the SVM classifier. To extract the discriminative features, Zhang et al. [32] used Partial Least Squares Regression (PLSR) to reduce the dimension of the scaled spectrograms. Afterword, SVM was used with the extracted features for classification. The proposed method was able to differentiate heart murmur from extrasystole with precision reached 91% using two public datasets offered by the PASCAL classifying heart sounds challenge. Another research study from the same authors Zhang et al. [49] proposed a method to analyze the heart signals based on scaled spectrogram and tensor decomposition. They used the following steps: (1) scaling the heart signal spectrograms into a defined size; (2) reducing the dimension of the scaled spectrograms; (3) extracting the intrinsic structure of the scaled spectrograms using tensor decomposition method, and finally, (4) classifying the heart signals using SVM and extracted features. The proposed method is evaluated on PASCAL and 2016 PhysioNet challenge, and the highest normal precision was 96%.

Redlarski et al. [50] combined SVM and modified cuckoo search algorithm with linear predictive coding (LPC) coefficients as input feature to build heart sounds diagnostic system. The developed system achieved accuracy of 93% for separating innocent murmur (S1, S2, S3, and S4) and organic murmur. Güraksin and Uguz [51] proposed the use of Least-squares SVM (LS-SVM) for heart sound signal classification. The wavelet Shannon entropy feature vectors were extracted and inputted to the classifier. A classification accuracy of 96.6% was obtained using their proposed technique. Patidar and Pachori [52] reported a method for cardiac sound signals features extraction using constrained tunable-Q wavelet transform (TQWT). LS-SVM was used then for classification with various kernel functions. An classification accuracy of 94.01% was registered using their proposed model.

Other research studies proposed the use of KNN algorithms to classify abnormal heart sounds. Oliveira et al. [53] utilized KNN algorithms to detect cardiac murmurs using a combination of time-frequency domain and perceptual and fractal analysis. Hamidi et al. [54] suggested two techniques to distinguish between normal and abnormal heart sound signals. In the first proposed technique, the power spectrum for the fitted signal curve was calculated and used as the first feature. In the second technique, the cardiac signal was divided into segments and the fractal dimension was calculated for each segment then the resultant signal was considered as another feature. Both features were used as inputs into KNN classifier and an overall accuracy of 92%, 81% and 98% were achieved, respectively, for three used datasets.

Potes et al. [55] used both the Adaboost and Convolutional Neural Network (CNN) classifiers to classify the heart sounds into normal and abnormal for the PhysioNet/CinC Challenge 2016. A group of time-frequency extracted features was used for PCG signal classification and their accuracy was 86%. A study by Bozkurt et al. [56] suggested the use of MFCC, Mel-Spectrogram, and sub-band envelopes features to automatically detect heart abnormality from PCG signal. They reported 81.5% accuracy, 78.5% specificity, and 84.5% sensitivity detection rate after inputting the proposed features into the CNN learning algorithm.

Messner et al. [57] detected the positions of S1 and S2 in heart sound signals using deep recurrent neural network (D-RNN) along with spectral and envelope features. They used virtual-adversarial training (VAT) dropout and data augmentation for regularization. They achieved an average score of F1 around 96% on an independent test set. Yaseen et al. [58] proposed heart sound automatic classification based on several extracted features. MFCC and DWT were used to extract the features of heart sound signals. While for classification, deep neural network (DNN), SVM, and centroid displacement-based KNN were selected for the classification stage. Their proposed methodology was proven to diagnose heart disorders in patients with 97% accuracy.

Chen et al. [59] used regression tree-based classification scheme with a CWT to differentiate organic from functional murmurs. They reported 90% classification accuracy in their research paper. For feature extraction, SVD and QR-Factorization were used on the time-frequency matrix attained using the CWT. In addition, features based on Gini index and the Shannon entropy were calculated as well on the decomposition process. To reduce the computational complexity, only number of features was selected using the Sequential Forward Floating Selection (SFFS) algorithm for the classification system.

Safara et al. [60] used BayesNet classifier to identify cardiac valve disorders, and they reached 96% classification accuracy. New wavelet packet entropy feature was introduced in their research paper to classify of five types of heart sounds and murmurs. Wavelet packet transform was employed for heart sound analysis, and the entropy was calculated for deriving feature vectors.

Guillermo et al. [61] proposed a Radial Wavelet Neural Network (RWNN) with Extended Kalman Filter (EKF) model for heart disease classifications. CWT was used to segment PCG signal and identify primary heart sounds, S1 and S2. The dimensional features that were extracted from the cardiac cycles are then used as inputs into the proposed model. They reported 98.04% classification accuracy rate using the proposed learning model.

Safara et al. [62] considered the use of multilevel basis selection (MLBS) method for signals with a small range of frequencies. Their method based on preserving only the most useful bases of a wavelet packet decomposition tree through applying the following elimination criteria: frequency range, noise frequency, and energy threshold. In classifying heart sounds, an accuracy of 97.56% was achieved using the MLBS method.

Thiyagaraja et al. [63] presented patient-centered device system that can monitor patient’s cardiac status. The reported system helps on recording, processing, and classification heart sounds signals. In their system, they used both MFCC and HMM for heart signals classification into normal/murmur with accuracy of 92.68%.

Choi et al. [64] proposed to segment the cardiac spectral using multi-Gaussian (MG) fitting technique to detect abnormal heart sounds. The following measurements of the Gaussian peaks: spectral profiles, maximum frequency, amplitude, half-width, area portion, and loss of area were examined to segment the cardiac spectral curve of different heart sounds.

In another work proposed by Varghees and K.I. [65], the PCG signal was initially decomposed by the experimental wavelet transform (EWT). The boundaries of the heart sounds were detected using the Shannon entropy and instantaneous phase. The accuracy results for the proposed system was 91.92%.

Choi et al. [66] proposed the use of wavelet packet (WP) technique for heart sounds analysis. They use the upper-limit peak frequency, the WP coefficient position related to the upper-limit peak frequency, and the wavelet energy fractions and entropy information features to detect the heart murmurs. Their murmur detection method yielded a classification efficiency of 99.78% specificity and 99.43% sensitivity.

In 2012, Xiefeng et al. [67] used a family of wavelets to develop their model, after that, they extracted features of heart sounds by using of the heart sounds linear band frequency cepstral (HS-LBFC). For heart sound identification, they used the similarity distance method.

Abo-Zahhad et al. [68] introduced an approach for human recognition using heart sounds. The proposed method is based on adopting wavelet packet cepstral coefficient (WPCC) as features for heart sound signal identification. The proposed features employ a nonlinear wavelet packet filter banks that were constructed to match the acoustic nature of the heart sound. After evaluated against an open dataset HSCT-11, their proposed method reported 91.05% classification accuracy.

## 3. The Proposed Model

**Figure 1:**
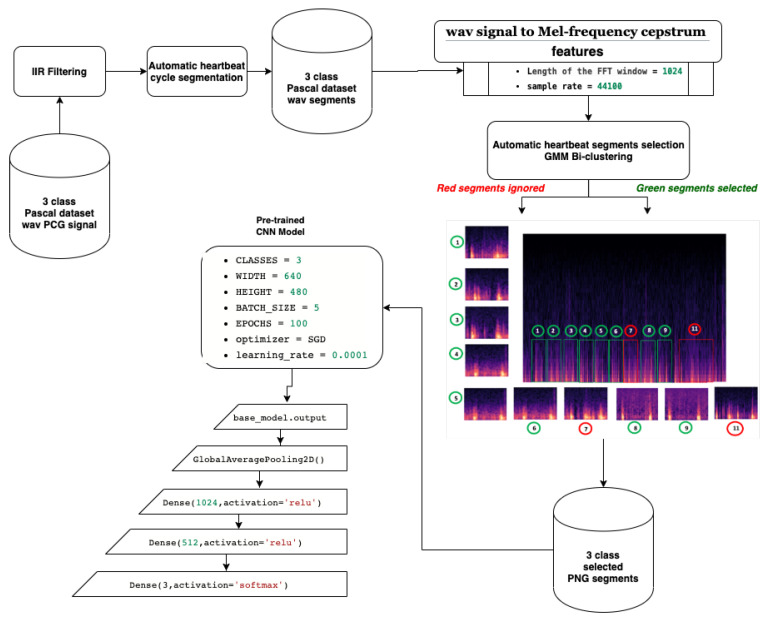
Proposed heart sound detection model.

In this paper, a method that combines both supervised and unsupervised learning approaches was developed. The proposed model implements a classification approach that enables the recognition of both normal and abnormal heartbeat rhythms. Figure 1 gives a general overview of the proposed model. In the next subsections, we explain each step in more detail.

### 3.1. Preprocessing

In this paper, the preprocessing step comprises four parts, namely, denosing, automatic heart cycle segmentation, Mel-Frequency spectrum images, and segment selection by clustering.

#### 3.1.1. Noise Filtering

**Figure 2:**
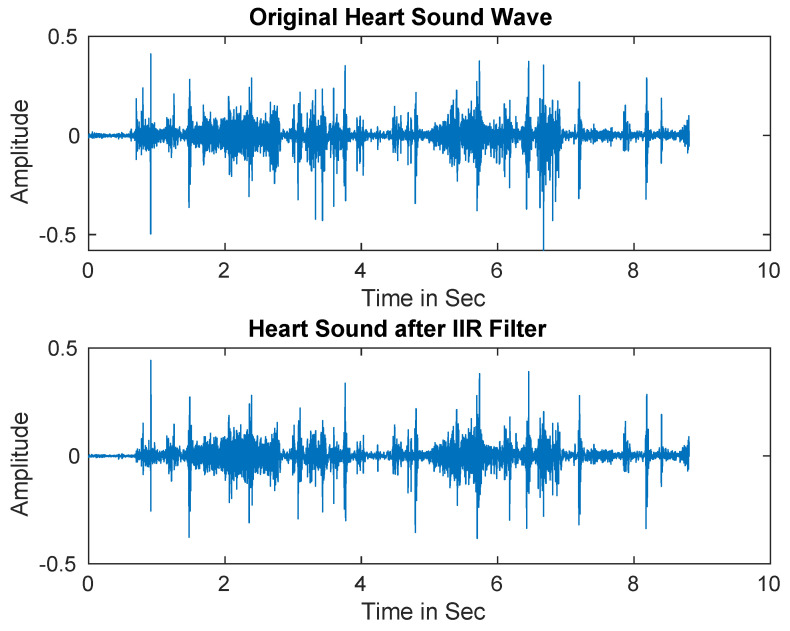
Heart sound signals after applying Infinite Impulse Response (IIR) filter.

In practice, PCG signals are often corrupted by different types of noise that may decrease the detection accuracy. Therefore, IIR filter was first utilized to separate the noise from the signals [69]. Figure 2 shows the original heart sound signal versus the denoised signal.

#### 3.1.2. Automatic Heart Cycle Segmentation

After IIR filtering, we proceed with heart cycle segmentation. Firstly, signals were downsampled to 2 kHz since most low heart sound signal frequency is 25–120 Hz, whereas our signal sampling frequency was 44.1 kHz. Then signals were normalized according to Equation (1).

(1) $$NS\left(t\right)=\frac{S(t)}{max(|S(t)|)}$$

where NS(t) and S(t) denote the normalized heart signal and the original heart signal, respectively.

After that, we performed envelope detection using Daubechies wavelet decomposition. To get low frequency signals, we computed adaptive threshold using wavelet decomposition coefficients *C*, thr=μ(C)+f*σ(C). After calculating adaptive threshold, we set wavelet decomposition coefficients smaller than threshold and larger than threshold assign as zero as seen in Equation (2).

(2) $$\tilde{c_{i}}=\left\{\begin{array}{cc} c_{i}, & \mathrm{if}\;c_{i}<thr.\\ 0, & \mathrm{otherwise}. \end{array}\right.$$

where $c_{i}$ is wavelet decomposition coefficient.

**Figure 3:**
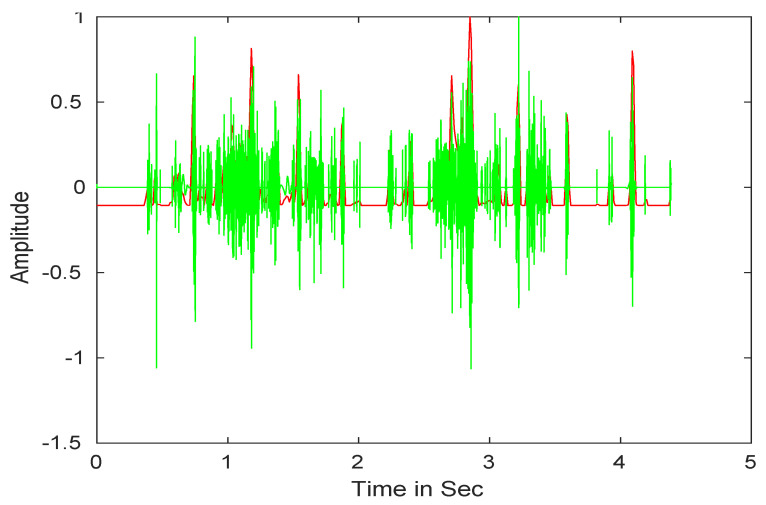
Heart sound signals envelope detection.

After that, we performed the wavelet reconstruction to extract the low-frequency heart sound. Finally, we computed Shannon entropy (see Equation (3)), then, the average Shannon entropy is standardized as seen in Equation (4) [70]. The envelope of input signals is shown in Figure 3.

(3) $$SE\left(t\right)=-\frac{1}{N}\sum_{j=1}^{N}LS\left(j\right)\log LS\left(j\right)$$

where LS(j), *N* and SE(t) denote the low-frequency heart sound segment, the number of signal samples per segment, and the Shannon entropy, respectively.

(4) $$NLS_{t}=\frac{SE\left(t\right)-\mu_{t}}{\sigma_{t}}$$

where $NLS_{t}$ is the the normalized Shannon energy, $\mu_{t}$ is the mean of energy SE(t) of the signal *t*, and $\sigma_{t}$ is the standard deviation of energy SE(t) of the signal *t*.

**Figure 4:**
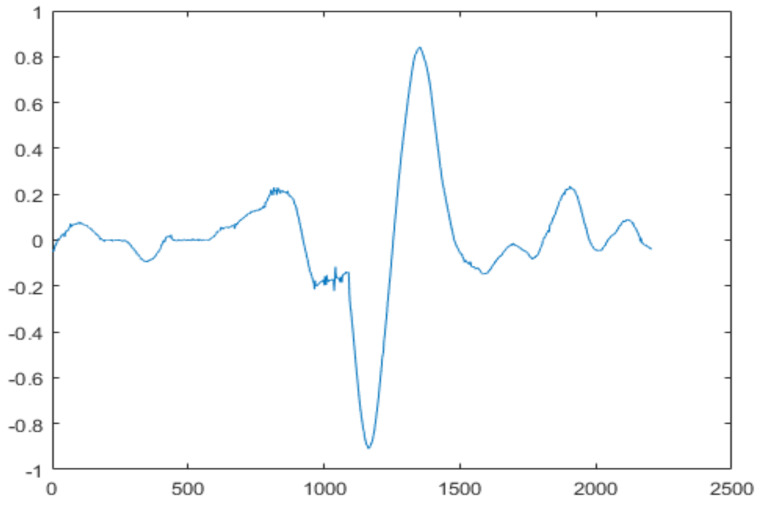
A single heart cycle segment.

The final step is to identify the heart sound segments. Given the semiperiodic nature of heart sounds, this step can be accomplished more efficiently if the cardiac cycle is calculated. In this study, we used a cardiac cycle calculation approach based on the unbiased autocorrelation function (UACF) [70,71]. After defining the cardiac cycle, the components of the sound of the heart can be identified and segmented. A single heart cycle segment is shown in Figure 4.

#### 3.1.3. Mel-Frequency Spectrum Images

**Figure 5:**
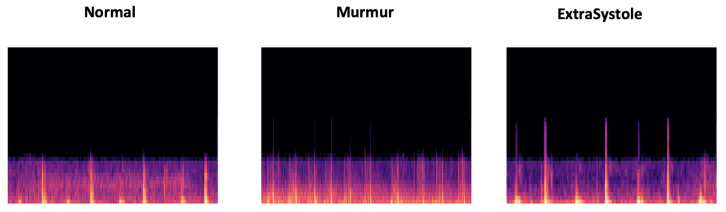
Overview of extrasystole-mumur-normal MFCC features represented in PNG images.

**Figure 6:**
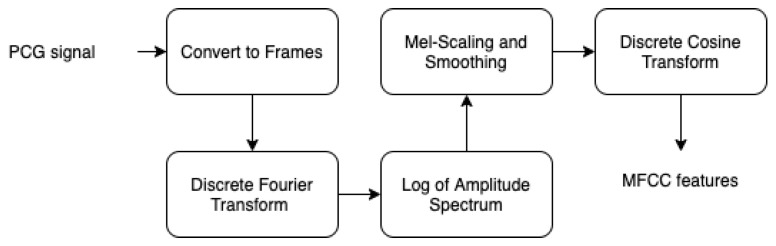
MFCC steps.

MFCC is considered as a powerful acoustic feature extractor generating essential information from any audio signal. This technique proved its robustness especially in speech recognition field Dave [72], Han et al. [73], Al Marzuqi et al. [74] through the ability to represent the signal amplitude spectrum in a compact form. In our case, we used MFCC technique for the aim to extract PCG spectrum features to be stored in PNG image (see Figure 5). In fact, Figure 6 shows the different processing steps related to MFCC:

1. By performing a Hamming windowing at fixed interval of 1024 (in our case), the PCG signal is divided into acoustic chunks. The outcome of this step is a vector representing the cepstal features related to each chunks.
2. Applying discrete Fourier transform (DFT) to each window chunk.
3. For each DFT chunk, it retains only the amplitude spectrum logarithm to conserve the signal loudness property, which was found to be approximately logarithmic.
4. To obtain essential frequency features, MFCC technique is based on spectrum smoothing process.
5. By applying discrete cosine transform to the fourth step output, we obtain the MFCC features of our PCG signal.

#### 3.1.4. Segment Selection by Clustering

The main objective of our heartbeat segmentation method is to divide PCG signal into different heartbeat cycles with the aim of improving CVD recognition. However, it is well-known that PCG signal is very noisy, which means we can find noise even in one or multiple heart cycle segments. Therefore, the CVD training process is affected by this constraint, causing a CVD signature extraction failure. The idea behind our segment selection method is to apply clustering technique to eliminate the undesired segments; those that influence on the recognition result. We start with the hypothesis that the majority of obtained heart cycle segments are correlated and contain less noise, which means it could be adopted for CVD signature extraction. Firstly, we proceed to a biclustering by applying a parametric clustering method. Then, we ignore the cluster having the minimal number of segments (noisy segments). In other words, the segment selection process are based on the segments belonging to the bigger cluster.

We chose to use mixture Gaussian model (GMM) [75], which is a parametric unsupervised clustering method. This method could be used for partitioning data into different groups according to the probabilities of belonging to each Gaussian. GMM is based on a mixture of Gaussian’s relying on learning the laws of probability that generated the observation data $x_{n}$ (See Equation (5)).

(5) $$\begin{array}{c} f\left(x_{n}|\theta_{k}\right)=\sum_{k=1}^{M}\pi_{k}N\left(x_{n}|\mu_{k},\sigma_{k}^{2}\right) \end{array}$$

With $N\left(x_{n}|\mu_{k},\sigma_{k}^{2}\right)=\frac{1}{{\left(2\pi \right)}^{d/2}\sigma^{1/2}}e^{\left(-\frac{1}{2\sigma_{k}^{2}}{\left(x_{n}-\mu_{k}\right)}^{2}\right)}$, $\pi_{k}\in 1\ldots M$: the probability of belonging to a Gaussian k with k∈1…M ), $\mu {}_{k}\in 1\ldots M$: the set of the M Gaussian averages, $\sigma_{k}^{2}\in 1\ldots M$: the set of covariances matrices and $\theta_{k}=\pi_{k},\mu_{k},\sigma_{k}^{2}$. Similarly, the multidimensional version of the Gaussian is as follows: $N\left(x_{n}|\mu_{k},\Sigma_{k}\right)=\frac{1}{{\left(2\pi \right)}^{d/2}\Sigma^{1/2}}e^{\left(-\frac{1}{2}{\left(x_{n}-\mu_{k}\right)}^{T}-\Sigma_{k}^{-1}\left(x_{n}-\mu_{k}\right)\right)}$. The best-known method for estimating the GMM parameters ($\pi_{k},\mu_{k}$ and $\sigma_{k}^{2}$), is the iterative method of maximum likelihood calculation (expectation-maximization algorithm or EM [76]). The EM algorithm could be defined through 3 steps:

- Step 1: Parameter initialization $\theta_{k}:\pi_{k},\mu_{k},\sigma_{k}^{2}$
- Step 2: Repeat until convergence
  • Estimation step: calculation of conditional probabilities $t_{ik}$ that the sample i comes from the Gaussian k. $t_{\left(i,k\right)}=\frac{\pi_{k}N\left(x_{i}|\mu_{k},\sigma_{k}^{2}\right)}{\sum_{j=1}^{m}\pi_{k}N\left(x_{i}|\mu_{j},\sigma_{j}^{2}\right)}$ with j∈1,…,m: the set of Gaussians.
  • Maximization step: update settings $\theta_{k}^{estim}=argmax_{\theta_{k}}\left(\theta_{k},\theta_{k}^{old}\right)$ and $\pi_{k}^{estim}=\frac{1}{n}\sum_{i=1}^{N}t_{i,k}$, $\sigma_{k}^{2estim}=\frac{\sum_{i=1}^{N}t_{i,k}{\left(x_{i}-\mu_{k}^{estim}\right)}^{2}}{\sum_{i=1}^{N}t_{i,k}}$, $\mu_{k}^{estim}=\frac{\sum_{i=1}^{N}t_{i,k}x_{i}}{\sum_{i=1}^{N}t_{i,k}}$

The time complexity of EM algorithm for GMM parameters estimation McLachlan and Peel [75], McLachlan and Krishnan [76], Bishop [77], Hastie et al. [78], is as following: If *X*: is the dataset size, *M*: the Gaussian number and *D*: the dataset dimension.

EM Estimation step O(XMD+XM).

EM Maximization step O(2XMD).

### 3.2. CNN Classification

The technological progress of deep learning paved the way for boosting the use of computer vision, especially by using CNN. Much research was conducted to recognize objects [79], speech emotion [80], gestures [81], or even visual speech recognition [82]. In fact, CNN using transfer learning techniques was extremely exploited [83,84,85,86], especially when it comes with a small training set. Due to the lack of publicly available big training set of labeled PCG signals, we chose to adopt CNN transfer learning technique [87]. By fine-tuning the existing pretrained CNN models that were already trained on ImageNet, we can just train our model on new classification layer. After applying the different preprocessing steps presented in Figure 1 on pascal PCG dataset, we obtain a set of PNG images containing visual representation of MFCC features that are trained by our fine-tuned CNN model.

**Figure 7:**
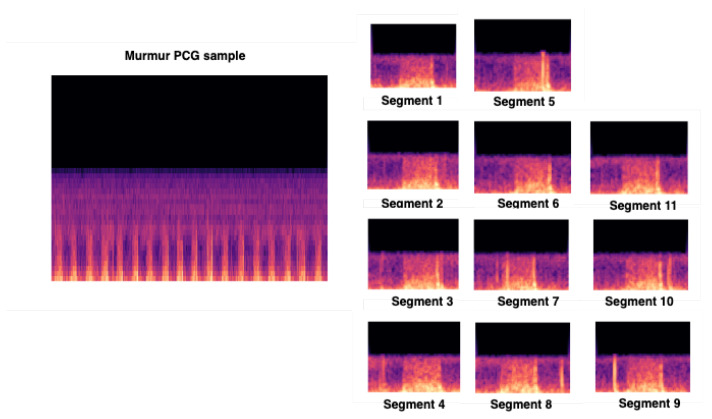
Overview of our CNN input training images issued from preprocessing steps.

We used CNN input shape equals to (480, 640, 3), and we conserved the pretrained convolutional layers used for feature extraction. We proceeded to fine-tuning by adding 4 layers. For a better feature vector representation, we added GlobalAveragePooling2D, which uses a parser window moving across the feature matrix and pools the data by averaging it (to take the corner cases into the account). Then, we added two dense layers, respectively, 1024 and 512, to allow learning more complex functions, and therefore, for better classification results. To be able to classify the results, we added dense layer, with Softmax as activation function. Figure 7 gives an overview of the input training images segments.

## 4. Performance Evaluation

In this section, we first present the experimental setup. Secondly, the used dataset is explained.

### 4.1. Experimental Setup

In our pretrained CNN experimental setup, we preserved all the convolutional layers related to all the used Keras pretrained models and we added 4 layers as described in the section (CNN classification). We used Stochastic gradient descent optimizer for weight update with learning rate = 0.0001 and Keras default momentum, batch size = 5 and epochs = 100.

The CNN training process was performed on Google Colab platform allowing the use of a dedicated GPU: 1xTesla K80, having 2496 CUDA cores, compute 3.7, 12 GB (11.439 GB Usable) GDDR5 VRAM. Table 1 presents the details related to the different Keras Pretrained CNN models used in this work.

### 4.2. Dataset

Our work is based on the publicly available pascal Bentley et al. [88] and Physionet datasets [89]. As shown in Table 2, which summarizes the structure of this dataset, we used 231 samples obtained by merging the Normal samples from training set A and training set B without considering Btraining_noisynormal (samples). Concerning the Murmur class, we merged 34 samples from training set A with 95 samples issued from merging 66 samples from training set B and 29 samples from noisy_murmur folder. Considering Extrasystole class, we relayed on 65 samples issued from merging 19 samples from training set A and 46 samples from training set B. Concerning PhysioNet [89] dataset, it contains 665 normal samples, and 2575 abnormal samples in WAV format, and the majority of PCG samples are concentrated in the duration range between 8 and 40 s for normal and abnormal class.

In fact, after performing the preprocessing step, we obtained a set of PCG samples (heart cycle) that represent the selected heart cycles. These PCG heartbeat cycles are then transformed into PNG images to be trained by our CNN models. As shown in Table 3, our segment selection process selects only the segments having close MFCC features and ignores the others. For example, 323 of Normal PCG segments are selected and 33 are ignored from a total of 356 PCG segments. Except the Extrasystole class, we notice that the training set size of Normal and Murmur class increases. The total number of Normal class samples goes from 231 to 323 samples; Murmur goes from 129 to 317 samples, and Extrasystole goes from 65 to 62 samples. In other words, the CNN model is trained only on heart cycle segments and not on the overall PCG signal.

## 5. Results and Discussion

In this section, we present and discuss our experimental results. The main objective behind this experimental study is to analyze the effect of the segment selection process on the classification results. After performing our preprocessing steps, we experimented 17 Keras pretrained CNN models with and without the use of our segment selection process.

**Figure 8:**
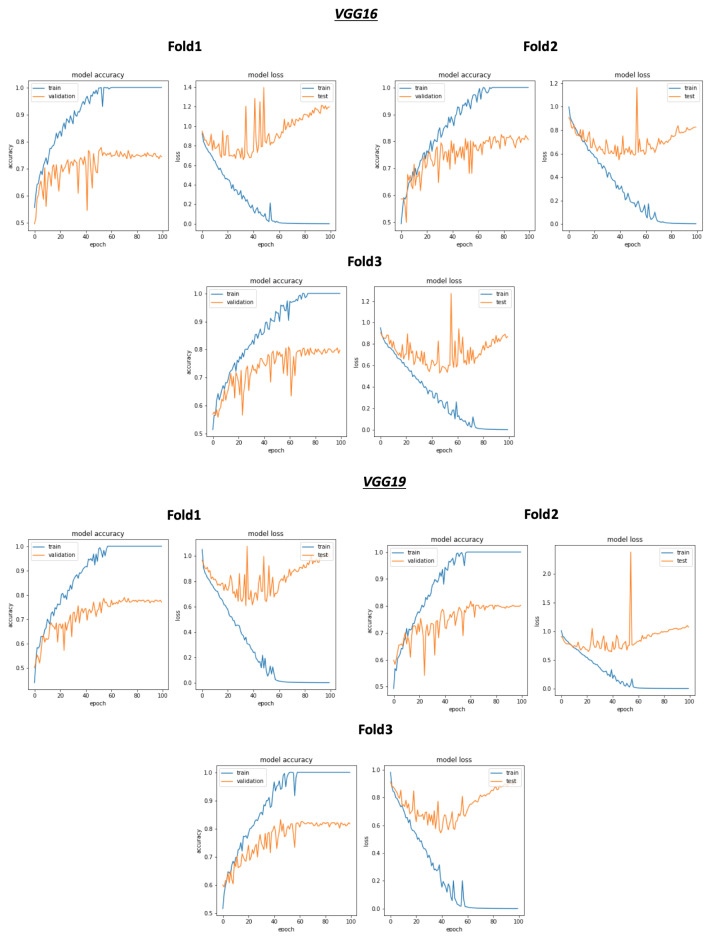
Overview of CNN VGG16-VGG19 validation accuracy curve without selection process.

**Figure 9:**
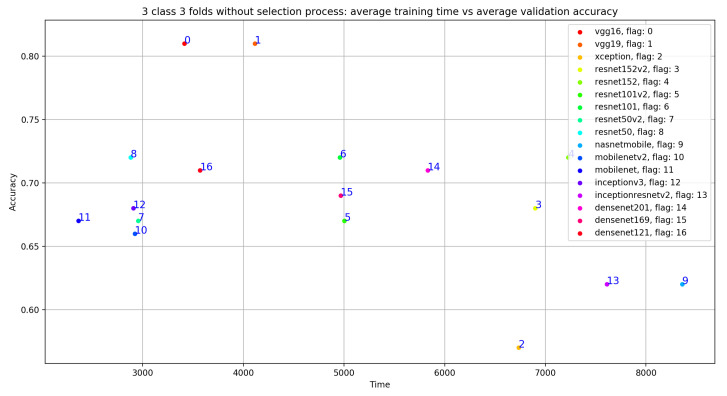
Overview of CNN models average training time vs average validation accuracy without selection process.

As shown in Figure 8 and Table 4, the best average validation accuracy = 0.81 is obtained using VGG16 and VGG19 through 3 cross validation folds. The training time plots seen in Figure 9 gives us an idea about the VGG16 and VGG19 ranking, which is respectively VGG16_rank = 6 and VGG19_rank = 9. By using Fold1, VGG16 and VGG19 reached their best validation accuracy respectively in Epoch 55 and Epoch 58. Considering Fold2, respectively in Epoch 80 and Epoch 62, VGG16 and VGG19 reached their best validation accuracy, and using Fold3, VGG16 and VGG19 reached their validation accuracy peaks in Epoch 60 and Epoch 48, respectively. Concerning TPR results, VGG19 reached the best average TPR = 0.73 value (as seen in Table 5).

**Figure 10:**
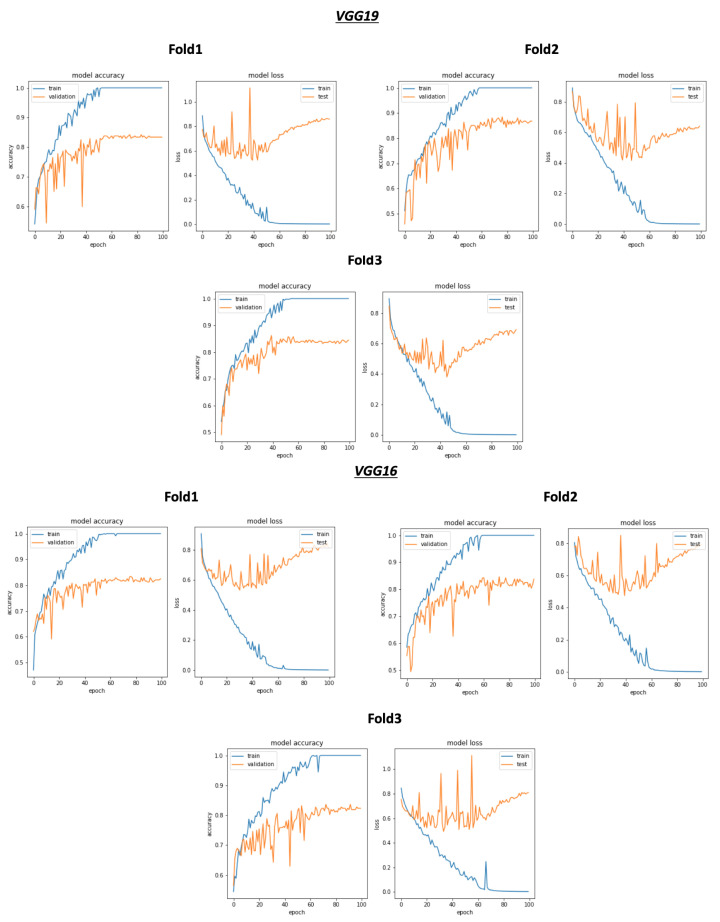
Overview of CNN VGG16-VGG19 validation accuracy curve with selection process.

Concerning the classification results using the selection process, there is a significant improvement in the average validation accuracy and the average TPR results. As seen in Figure 10, Table 6 and Table 7, the best validation accuracy average and TPR average are obtained using VGG19. The validation accuracy average and TPR average improvement in VGG19 respectively goes from 0.81 to **0.87** and from 0.73 to **0.83**. In other words, the additional three convolutional layers for VGG19 depth = 26 (as seen in Table 1), compared to the depth = 23 for VGG16, have a direct impact on the validation accuracy related to this configuration. Despite the deep architecture used in DenseNet201 with a number of layers equal to 201, we can see that the validation accuracy (as seen in Table 6) is equal to 0.75 but is less than VGG16 and VGG19, which argues that the depth of the model has a random impact on the validation accuracy.

**Figure 11:**
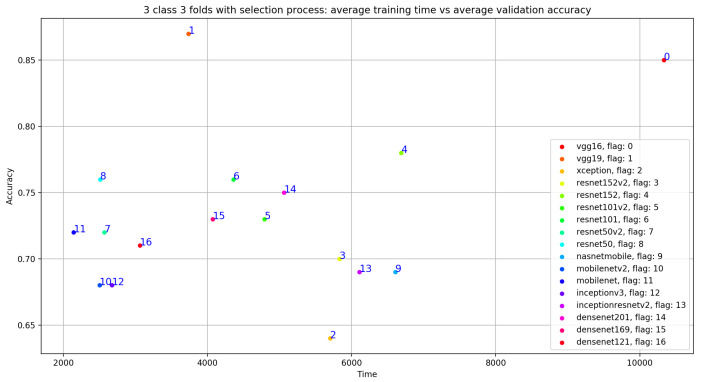
Overview of CNN models average training time VS average validation accuracy with selection process.

As shown in Figure 9, despite the same validation accuracy results without the use of the selection process, VGG16 requires less training time compared to that of VGG19. On the other hand, Figure 11 shows that by using the selection process, the training time of VGG19 is considerably less than VGG16 training time, which is the worst one compared to all the used models.

We also conducted a comparative study to compare our classification results with that of some recent related works that are based on Pascal 2011 Dataset. As seen in Table 8, except the work of Zhang et al. [32], the majority of these works don’t exploit the entire Pascal dataset samples. For example, in the work of Malik et al. [99], the authors used 31 signals. Similarly, Chakir et al. [100] relayed on 52 signal, Chakir et al. [101] exploited 14 signals from dataset A, and 127 from dataset B. Pedrosa et al. [41] used 111 signals, and in Sidra et al. [102] work, the authors relayed on 24 signal for normal class and 31 for abnormal class. This selection strategy can be explained by the fact that Pascal Dataset contains too much noisy signals (with background noise), which influences the classification results. The fact that we exclude the noisy signals means the classification result improves immediately, which explains the good results obtained by Malik et al. [99] with overall accuracy = 0.89, overall precision = 0.91, and overall TPR = 0.98. By applying our methodology on the totality of signals in Pascal dataset, we just select the useful heart cycle segments and ignore those with noise without ignoring the overall sample. Due to the use of our segmentation and selection process, we obtained more accurate classification results compared to that of Zhang et al. [32] and Balili et al. [103] works. Also, as seen in the Table 9, we obtained encouraging results in term of **micro_accuracy = 0.91**, **micro_sensitivity = 0.84**, **micro_precision = 0.84** and **micro_specificity = 0.92**.

**Figure 12:**
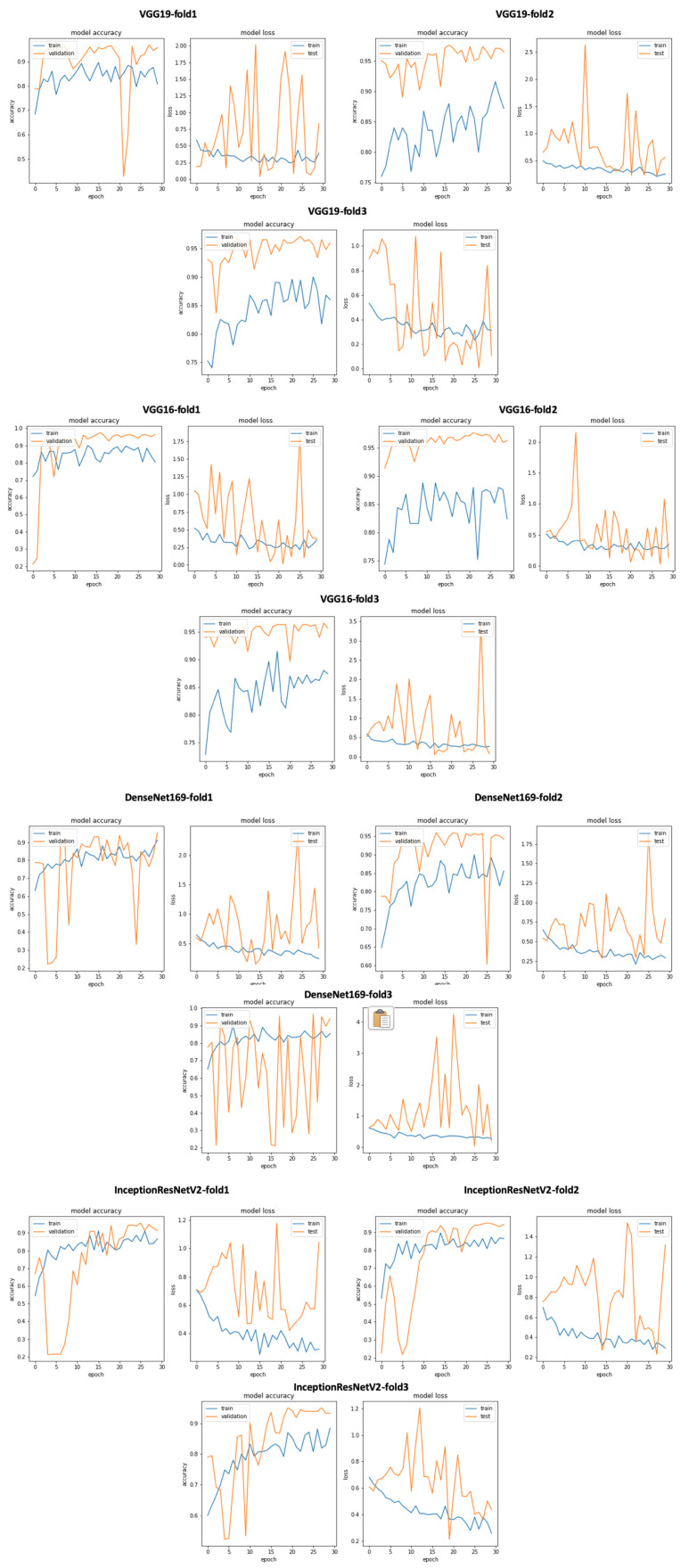
An overview of our approach using VGG19, VGG16, DenseNet169 and InceptionResNetV2 training and validation curves on PhysioNet dataset.

We experimented with our approach also on PhysioNet data set (two class dataset). We adapted the classification layer of all of the 17 CNN models to be able to recognize 2 classes (Normal and Abnormal). Figure 12 gives an overview of training and validation accuracy with model loss related to VGG19, VGG16, DenseNet169 and InceptionResNetV2. As seen in Table 10, VGG19 outperforms all the other Keras 16 models with excellent classification results: accuracy = 0.97, TPR = 0.946, Precision = 0.944 and Specificity = 0.946. On the other hand, we performed a comparative study with relevant state of the art approach summarized in Table 11. As seen in this table, we achieved excellent classification results with an accuracy equal to 0.97, a sensitivity equal to 0.946, a precision equal to 0.944, and specificity equal to 0.946.

## 6. Conclusions and Future Work

In this work, we presented an AI-based approach for automatic phonocardiogram (PCG) signal analysis to help in the preliminary diagnosis of different heart diseases. The discussed method is considered as a new cardiovascular disease recognition approach experimented on two PCG datasets: Pascal and PhysioNet. Firstly, we performed preprocessing steps through the use of infinite impulse response (IIR) filtering followed by a robust heart cycle segmentation technique. Secondly, we presented our segment selection process, which enables the automatic selection of the maximum correlated segments. Finally, we fine-tuned pretrained model to be trained on the heart cycle mfcc spectrogram images. We obtained encouraging classification results for both Pascal and PhysioNet datasets with overall accuracy 0.87, overall precision 0.81, and overall sensitivity 0.83 using Pascal, and accuracy 0.97, sensitivity 0.946, precision 0.944, and specificity 0.946 using PhysioNet dataset. To our knowledge, these results can be considered the best classification results compared to that of the majority of previous works, which relied on the entire PhysioNet and Pascal dataset signals. We plan to combine both mask RCNN for object detection and CNN models to improve the classification results based on models voting. 

## Figures and Tables

**Table 1:** Keras pretrained CNN models.

Model	Citation	Layers	Size	Parameters
**Xception**	Chollet [90]	71	85 MB	44.6 millions
**VGG19**	Simonyan and Zisserman [91]	26	549 MB	143.6 millions
**VGG16**	Simonyan and Zisserman [91]	23	528 MB	138.3 millions
**ResNet152V2**	He et al. [92]	-	98 MB	25.6 millions
**ResNet152**	He et al. [92]	-	232 MB	60.4 millions
**ResNet101V2**	He et al. [92]	-	171 MB	44.6 millions
**ResNet101**	He et al. [92]	101	167 MB	44.6 millions
**ResNet50V2**	He et al. [92]		98 MB	25.6 millions
**ResNet50**	He et al. [92]	-	98 MB	25.6 millions
**NASNetMobile**	Zoph et al. [93]	-	20 MB	5.3 millions
**MobileNetV2**	Sandler et al. [94]	53	13 MB	3.5 millions
**MobileNet**	Howard et al. [95]	88	16 MB	4.25 millions
**InceptionV3**	Szegedy et al. [96]	48	89 MB	23.9 millions
**InceptionResNetV2**	Szegedy et al. [97]	164	209 MB	55.9 millions
**DenseNet201**	Huang et al. [98]	201	77 MB	20 millions
**DenseNet169**	Huang et al. [98]	169	57 MB	14.3 millions
**DenseNet121**	Huang et al. [98]	121	33 MB	8.06 millions

**Table 2:** Overview of pascal dataset structure.

Training Set	Class		
	Normal	Murmur	Extrasystole
**A**	31	34	19
**B**	200	95	46
**Total**	**231**	**129**	**65**

**Table 3:** Overview of selected PCG segments according to each class.

Training Set	Class Segments		
	Normal	Murmur	Extrasystole
**Selected**	**323**	**317**	**62**
**Ignored**	33	14	44
**Total segments**	**356**	**331**	**106**

**Table 4:** Validation accuracy of CNN models using 3 class 3 folds without segment selection.

Model	Accuracy			
	Fold1	Fold2	Fold3	AVG
**VGG16**	0.77	0.82	0.80	**0.81**
**VGG19**	0.78	0.81	0.83	**0.81**
**Xception**	0.56	0.58	0.58	0.57
**ResNet152V2**	0.66	0.69	0.68	0.68
**ResNet152**	0.73	0.73	0.71	0.72
**ResNet101V2**	0.66	0.67	0.69	0.67
**ResNet101**	0.69	0.72	0.74	0.72
**ResNet50v2**	0.68	0.69	0.64	0.67
**ResNet50**	0.72	0.73	0.72	0.72
**NasNetMobile**	0.63	0.62	0.60	0.62
**MobileNetV2**	0.68	0.67	0.63	0.66
**MobileNet**	0.66	0.67	0.67	0.67
**Inceptionv3**	0.68	0.68	0.68	0.68
**InceptionResNetV2**	0.59	0.66	0.61	0.62
**DenseNet201**	0.71	0.74	0.69	0.71
**DenseNet169**	0.68	0.70	0.70	0.69
**DenseNet121**	0.69	0.73	0.70	0.71

**Table 5:** Validation true positive rate (TPR) of CNN models using 3 classes (E: Extrasystole; M: Murmur; N: Normal) and 3 folds without selection process.

Model	TPR												
	Fold1				Fold2				Fold3				Avg
	E	M	N	Avg	E	M	N	Avg	E	M	N	Avg	
**VGG16**	0.36	0.77	0.90	0.68	0.62	0.80	0.89	0.77	0.31	0.83	0.93	0.69	0.72
**VGG19**	0.44	0.80	0.88	0.70	0.54	0.81	0.89	0.75	0.4	0.88	0.91	0.73	**0.73**
**Xception**	0.0	0.52	0.77	0.43	0.0	0.41	0.91	0.44	0.0	0.46	0.88	0.44	0.44
**ResNet152V2**	0.25	0.81	0.65	0.57	0.11	0.69	0.86	0.55	0.28	0.7	0.78	0.59	0.57
**ResNet152**	0.27	0.70	0.89	0.62	0.25	0.69	0.91	0.62	0.14	0.77	0.83	0.58	0.61
**ResNet101V2**	0.02	0.63	0.88	0.51	0.14	0.64	0.86	0.55	0.25	0.73	0.79	0.59	0.55
**ResNet101**	0.16	0.81	0.74	0.57	0.25	0.79	0.80	0.61	0.0	0.79	0.91	0.56	0.58
**ResNet50v2**	0.33	0.77	0.71	0.60	0.17	0.75	0.79	0.57	0.05	0.56	0.89	0.50	0.56
**ResNet50**	0.19	0.74	0.85	0.59	0.2	0.73	0.89	0.60	0.17	0.7	0.90	0.59	0.59
**NasNetMobile**	0.22	0.58	0.81	0.54	0.0	0.6	0.82	0.47	0.0	0.56	0.83	0.46	0.49
**MobileNetV2**	0.16	0.66	0.84	0.56	0.11	0.67	0.83	0.53	0.14	0.7	0.72	0.52	0.54
**MobileNet**	0.22	0.65	0.80	0.56	0.22	0.59	0.87	0.56	0.08	0.74	0.78	0.53	0.55
**Inceptionv3**	0.0	0.65	0.91	0.52	0.0	0.63	0.94	0.52	0.02	0.6	0.95	0.52	0.52
**InceptionResNetV2**	0.0	0.44	0.91	0.45	0.0	0.69	0.84	0.51	0.0	0.6	0.81	0.47	0.48
**DenseNet201**	0.25	0.69	0.88	0.60	0.34	0.73	0.86	0.64	0.17	0.73	0.81	0.57	0.60
**DenseNet169**	0.25	0.67	0.82	0.58	0.11	0.82	0.77	0.57	0.08	0.70	0.88	0.55	0.57
**DenseNet121**	0.19	0.72	0.83	0.58	0.4	0.72	0.84	0.65	0.17	0.79	0.78	0.58	0.60

**Table 6:** Validation accuracy of CNN models using 3 class 3 folds after segment selection.

Accuracy	Folds			
	Fold1	Fold2	Fold3	AVG
**VGG16**	0.85957	0.8383	0.84483	0.85
**VGG19**	0.84255	0.89711	0.86207	**0.87**
**Xception**	0.65106	0.61277	0.67241	0.64
**ResNet152V2**	0.72766	0.68287	0.69397	0.70
**ResNet152**	0.75745	0.74468	0.83621	0.78
**ResNet101V2**	0.75745	0.69787	0.73276	0.73
**ResNet101**	0.77447	0.74894	0.77155	0.76
**ResNet50v2**	0.72766	0.69362	0.73707	0.72
**ResNet50**	0.75745	0.73191	0.78017	0.76
**NasNetMobile**	0.69362	0.69787	0.68966	0.69
**MobileNetV2**	0.69787	0.65957	0.69397	0.68
**MobileNet**	0.74043	0.69787	0.71552	0.72
**Inceptionv3**	0.70638	0.68511	0.66379	0.68
**InceptionResNetV2**	0.69787	0.70213	0.67241	0.69
**DenseNet201**	0.77447	0.7234	0.76293	0.75
**DenseNet169**	0.74894	0.68085	0.75431	0.73
**DenseNet121**	0.7234	0.70213	0.72414	0.71

**Table 7:** Validation TPR of CNN models using 3 class (E: Extrasystole; M: Murmur; N: Normal) 3 folds with selection process.

Model	TPR												
	Fold1				Fold2				Fold3				Avg
	E	M	N	Avg	E	M	N	Avg	E	M	N	Avg	
**VGG16**	0.71	0.87	0.87	0.82	0.57	0.84	0.87	0.76	0.6	0.83	0.89	0.77	0.79
**VGG19**	0.80	0.83	0.86	0.83	0.57	0.89	0.93	0.80	0.9	0.87	0.84	0.87	**0.83**
**Xception**	0.0	0.59	0.83	0.47	0.0	0.39	0.94	0.44	0.0	0.61	0.85	0.48	0.47
**ResNet152V2**	0.61	0.72	0.75	0.69	0.62	0.71	0.72	0.68	0.75	0.68	0.69	0.70	0.70
**ResNet152**	0.80	0.66	0.83	0.77	0.42	0.73	0.81	0.65	0.6	0.83	0.87	0.77	0.73
**ResNet101V2**	0.66	0.69	0.83	0.73	0.09	0.63	0.87	0.53	0.7	0.73	0.73	0.72	0.66
**ResNet101**	0.61	0.73	0.84	0.73	0.47	0.76	0.78	0.67	0.7	0.80	0.74	0.75	0.72
**ResNet50v2**	0.47	0.66	0.84	0.65	0.19	0.61	0.87	0.55	0.4	0.72	0.81	0.64	0.62
**ResNet50**	0.66	0.78	0.75	0.73	0.28	0.77	0.77	0.61	0.45	0.75	0.86	0.69	0.68
**NasNetMobile**	0.23	0.57	0.89	0.57	0.0	0.78	0.75	0.51	0.3	0.66	0.78	0.58	0.55
**MobileNetV2**	0.28	0.61	0.86	0.58	0.09	0.55	0.87	0.50	0.3	0.54	0.91	0.58	0.56
**MobileNet**	0.76	0.61	0.86	0.74	0.38	0.95	0.50	0.61	0.6	0.8	0.65	0.68	0.68
**Inceptionv3**	0.0	0.63	0.91	0.51	0.0	0.68	0.81	0.50	0.15	0.69	0.72	0.52	0.51
**InceptionResNetV2**	0.0	0.65	0.87	0.51	0.0	0.75	0.78	0.51	0.0	0.63	0.83	0.48	0.50
**DenseNet201**	0.71	0.68	0.87	0.75	0.19	0.78	0.76	0.58	0.8	0.70	0.81	0.77	0.70
**DenseNet169**	0.76	0.78	0.71	0.75	0.33	0.82	0.61	0.58	0.45	0.69	0.86	0.67	0.67
**DenseNet121**	0.42	0.78	0.72	0.64	0.47	0.82	0.62	0.64	0.7	0.63	0.81	0.71	0.67

**Table 8:** An overview of our model results compared to that of some related works.

Works	PASCAL 2011 Signal Statistics	Classes	Overall Accuracy	Overall PPV	Overall TPR
Our method	Full labeled dataset	Normal, murmur, and extrasystole	**0.87**	**0.81**	**0.83**
Malik et al. [99]	31 signals	Normal, murmur, and other sounds	0.89	0.91	0.98
Chakir et al. [100]	52 signals	Normal and abnormal sounds	-	0.63	-
Zhang et al. [32]	Full dataset	Normal, murmur, and other sounds	-	0.67	-
Chakir et al. [101]	14 from A and 127 from B	Normal and murmurs	-	0.78	-
Balili et al. [103]	Full dataset	Normal, murmur, and other sounds	0.48	-	-
Pedrosa et al. [41]	111 signals	Normal heart sounds and murmurs	-	0.986	0.892
Sidra et al. [102]	24 normal and 31 abnormal	normal and abnormal	87.7	-	96.7

**Table 9:** Detailed average results of our model (VGG19) in terms of micro accuracy, micro TPR, micro precision, and micro specificity.

Folds	Accuracy			TPR (Sensitivity)			Precision (PPV)			TNR (Specificity)		
	Extra	Murmur	Normal	Extra	Murmur	Normal	Extra	Murmur	Normal	Extra	Murmur	Normal
**Fold1**	0.95	0.89	0.84	0.81	0.83	0.86	0.71	0.92	0.81	0.97	0.94	0.83
**Fold2**	0.96	0.92	0.89	0.57	0.90	0.93	0.92	0.92	0.85	0.99	0.94	0.86
**Fold3**	0.95	0.91	0.87	0.9	0.88	0.84	0.64	0.92	0.86	0.95	0.94	0.88
**Folds avg**	0.95	0.91	0.87	0.76	0.87	0.88	0.76	0.92	0.84	0.97	0.94	0.86
**Classes avg**	**0.91**			**0.84**			**0.84**			**0.92**		

**Table 10:** 3 Folds Average CNN test results using PhysioNet dataset.

Average	Accuracy	TPR (Sensitivity)	Precision (PPV)	TNR (Specificity)
**VGG16**	0.966	0.930	0.946	0.930
**VGG19**	**0.970**	**0.946**	**0.944**	**0.946**
**Xception**	0.828	0.877	0.732	0.877
**ResNet152V2**	0.824	0.873	0.730	0.873
**ResNet152**	0.490	0.667	0.640	0.667
**ResNet101V2**	0.438	0.665	0.422	0.665
**ResNet101**	0.690	0.592	0.812	0.592
**ResNet50v2**	0.698	0.736	0.728	0.736
**ResNet50**	0.620	0.763	0.685	0.763
**NasNetMobile**	0.203	0.489	0.350	0.489
**MobileNetV2**	0.228	0.497	0.526	0.497
**MobileNet**	0.671	0.679	0.673	0.679
**Inceptionv3**	0.659	0.791	0.686	0.791
**InceptionResNetV2**	0.863	0.908	0.765	0.908
**DenseNet201**	0.571	0.725	0.719	0.725
**DenseNet169**	0.493	0.675	0.606	0.675
**DenseNet121**	0.601	0.734	0.714	0.734

**Table 11:** Comparative analysis of our method with state-of-the-art methods using PhysioNet 2016.

Average	Accuracy	TPR (Sensitivity)	Precision (PPV)	TNR (Specificity)
**our approach**	**0.970**	**0.946**	**0.944**	**0.946**
[104]	0.8697	0.964	-	0.726
[55]	-	0.942	-	0.778
[105]	0.824	-	-	-
[106]	-	0.8095	-	0.839
[107]	-	0.84	-	0.957
[108]	0.852	-	-	-
[109]	-	0.885	-	0.921
[110]	0.879	0.885	-	0.878
[38]	0.97	0.932	-	0.951
[111]	0.915	0.983		0.846
[112]	0.892	0.90	-	0.884
[113]	0.88	0.88	-	0.87
[114]	0.85	0.89	-	0.816
[115]	0.826	0.769	-	0.883
[116]	0.801	0.796	-	0.806
[117]	0.9	0.93	-	0.9
[118]	0.79	0.77	-	0.8

## Data Availability

The study did not report any data.
